# Is it time to think about chronotherapy in migraine^☆^

**DOI:** 10.1016/j.neurot.2025.e00823

**Published:** 2025-12-23

**Authors:** Philip R. Holland, Rolf Fronczek

**Affiliations:** a Headache Group, Wolfson Sensory Pain and Regeneration Centre (WSPaRC), Institute of Psychiatry, Psychology and Neuroscience, King's College London, London, UK; b Department of Neurology, Leiden University Medical Center, Leiden, the Netherlands

**Keywords:** Migraine, Headache, Circadian, Chronotherapy

## Abstract

Migraine is among the most dynamic and disabling neurological disorders, affecting over one billion people globally. Attacks demonstrate rhythmic patterns of onset across the day and seasonally, highlighting that attack onset is influenced by an individual's biological rhythms. Indeed, an individual's chronotype (their endogenous circadian clock rhythm) predicts when they are most likely to have an attack. These biological rhythms are regulated by an endogenous master biological clock in the hypothalamus that coordinates the function of peripheral clocks, aligning biological processes and behaviours to environmental cues (e.g. daily rhythms in light-dark cycles). As such, circadian rhythms are essential to maintain normal neurological function and health, regulating the expression of approximately 50 % of all protein coding genes in mammals. Importantly, the majority of the World Health Organisations essential medicines and several migraine-related therapeutics directly target the products of these rhythmic genes or influence circadian-related genes directly. Therefore, the current review will focus on the potential for chronotherapy in migraine. Highlighting its potential to optimise the chronopharmacokinetic profile, therapeutic efficacy and reduce potential side effects of anti-migraine therapies. A greater understanding of which has the potential for significant impact, representing a low cost, scalable method to improve therapeutic response and inform a personalized therapeutic strategy.

## Introduction

Migraine and other selected primary headache disorders demonstrate varying degrees of daily (circadian [1,2]) and seasonal (circannual [3]) fluctuations in attack frequency, suggesting that attack susceptibility is broadly influenced by an individual's biological rhythm. These biological rhythms are regulated by the endogenous biological clock in the suprachiasmatic nucleus (SCN) of the hypothalamus, which acts to synchronize biological processes and behaviours in all mammals, considered a form of predictive homeostasis. Most notably the alignment of the 24-h sleep-wake cycle to the daily light-dark cycles ensures that physiological processes are aligned with predictable daily cycles. As such, a robust circadian clock is crucial to maintain normal neurological function and health [4]. Dysregulation of the body clock is linked to several neurological conditions, ranging from neurodegeneration to migraine and cluster headache [1,5]. In mammals, approximately 43 % of all protein coding genes demonstrate circadian rhythms in their transcription and the majority of the World Health Organisations essential medicines directly target the products of these rhythmic genes [6]. Therefore, the goal of chronotherapy is to harness these endogenous fluctuations, considering the body's circadian rhythms to optimise their chronopharmacokinetic profile, therapeutic efficacy and reduce potential side effects. The benefits of which are clear, for example, in cancer biology it is established that early day immuno-chemotherapy is associated with improved clinical outcome, overall survival and reduced side effects [7]. Alternatively, epileptic seizures often demonstrate a rhythmic onset pattern, and several studies have explored the potential of chronotherapy, demonstrating that therapies including carbamazepine and valproate are more effective when the administration is tailored to the individual's seizure chronotype [8,9], e.g. early morning or late evening. Building on the emerging role for chronobiology, chronopharmacokinetics and chronotherapy, the current review will focus on the growing evidence of the importance of circadian rhythms in migraine and the potential benefits of integrating chronotherapy to optimise therapeutic outcomes.

**Fig. 1:**
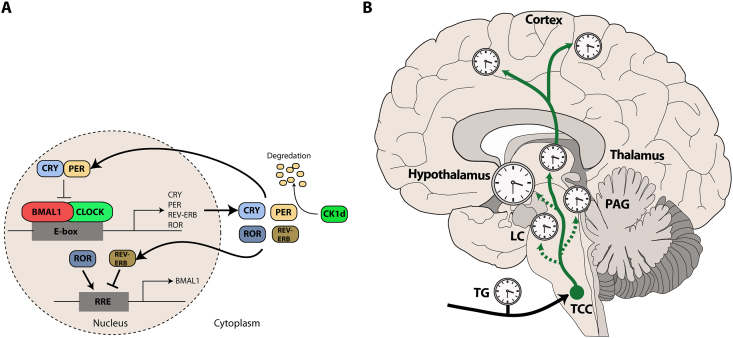
**Migraine and biological rhythms.** Circadian rhythms are generated by a core transcriptional-translational feedback loop. **(A)** Circadian Locomotor Output Cycles Kaput (CLOCK) and Brain and Muscle ARNT-Like 1 (BMAL1) heterodimerize in the cell nucleus where they bind to E-boxes in the promotor region of cryptochromes (CRY), period proteins (PER) and clock-controlled genes (retinoic acid-related orphan receptors (ROR) and REV-ERB) that increases their expression. The PER/CRY complex then binds to BMAL1/CLOCK to inhibit *Per/Cry* transcription. REV-ERB/ROR also modulates *Bmal1* transcription via the ROR response element (RRE). Casein Kinase 1 delta (CK1d), a key enzyme that has been linked to migraine further regulates the length of circadian rhythms via the phosphorylation of PER. **(B)** The pathophysiology of migraine involves several key structures that contain intrinsic clocks. The cell bodies of trigeminal sensory afferents arise in the trigeminal ganglion (TG) which innervate peripheral structures and project centrally to the trigeminocervical complex (TCC). From here, 2nd order neurons project to the thalamus and hypothalamus (which includes the master circadian clock in the suprachiasmatic nucleus), either directly or indirectly via key brainstem nuclei (e.g. the arousal promoting locus coeruleus (LC) and the periaqueductal grey (PAG). Information is then projected to several cortical regions. Clocks represent key migraine-related nuclei with intrinsic circadian clocks and rhythmic functions.

Circadian rhythms in all bodily tissues, including the SCN are generated by a core transcriptional-translational feedback loop (TTFL) of key circadian proteins, mainly oscillations in Circadian Locomotor Output Cycles Kaput (CLOCK) and Brain and Muscle ARNT-Like 1 (BMAL1) that activate Period (PER) and Cyptochrome (CRY). In Brief, CLOCK and BMAL1 form heterodimers that activate the transcription of PER (1–3) and CRY genes by binding to their E-box promoter sequences. In turn, PER and CRY then relocate to the nucleus and form a complex that ultimately the transcriptional activity of CLOCK and BMAL1 (Fig. 1A) [10]. This negative feedback loop takes about (“circa”) a day (“dies”) and permits the restarting of the cycle. Disruption of this rhythmic expression of core-clock and clock regulated genes (e.g. Casein Kinase 1 Delta (CK1δ)) is associated with several neurological disorders including migraine [11,12]. The biological clock is entrained by several external and internal signals, termed zeitgebers (time-givers), the most powerful of which in mammals is light [13]; however, food, exercise and other regular activities can influence our biological rhythms [14]. Light, signalling via intrinsically photosensitive ganglion cells (ipRGCs) in the eye, that express melanopsin [15] transmit entraining signals via the retinohypothalamic tract to the SCN, where it promotes the expression of core circadian clock genes including PER1 and PER2, resetting the TTFL [13]. Further, seasonal changes in day length (photoperiod) entrain the molecular clock to changing seasons, with resultant impacts on the circadian rhythm of gene expression and protein levels, altering physiological processes including hormone secretion (e.g. melatonin). While the SCN is considered the master biological clock, non-SCN, or peripheral clocks regulate semi-independent cellular circadian rhythms in multiple tissues throughout the body, that are synchronised by the SCN, but can act independently and be influenced by alternate cues, several of which are relevant for migraine (e.g. altered feeding patterns).

## Migraine and Circadian Rhythms

While there is variable data, a recent meta-analysis identified that over 50 % of people with migraine demonstrate circadian patterns of attack onset, with a preponderance for increased attack occurrence in the morning [1]. While seasonal rhythms suggest increased attack frequency in the Spring and Autumn [1], independent of established hormonal based rhythms [16]. In agreement, a person's chronotype, representing their endogenous circadian clock rhythm, differs between people with migraine and controls, with those with migraine less likely to have a normal chronotype, and presenting with more extreme early (early-bird) or late (night-owl) chronotypes [2]. The increased preponderance for an early chronotype coincided with increased early morning attack onset, while those people with migraine who present with a late chronotype tended to have increased attack onset later in the day, suggesting a clear link between an individual's endogenous biological clock rhythm and individual attack timing [2]. It is also established that people with migraine present with an increased vulnerability to attack onset following alterations to rhythmic behaviours. This is most prominently observed in the reported association between altered sleep-wake routines under circadian regulation, and increased attack risk [17]. Similarly, feed timing is considered an important zeitgeber, especially in metabolic tissues like the liver [18] and many patients report skipping meals as attack triggers [[19], [20], [21]].

Genetically, loss of function of CK1δ, a key enzyme the regulates the phosphorylation of PER proteins and regulates the speed of the molecular clock, leading to a familial advanced sleep syndrome [12], has been associated with migraine with aura [11]. Individuals from two families identified present with an advanced sleep phase (extreme morning larks) and an extremely high prevalence of migraine with aura [22]. In agreement, transgenic mice harbouring the human CK1δ mutation demonstrate a selective vulnerability to a lower dose of the clinical migraine trigger, nitroglycerin and a reduced threshold for cortical spreading depression [11], considered the underlying mechanisms of migraine aura [22]. Thus, these transgenic mice demonstrate a selective vulnerability to migraine-triggers when compared to wildtype littermate controls, mirroring the selective vulnerability of people with migraine when compared to healthy controls [23]. More generically, limited attempts to link more common forms of migraine to circadian gene disruptions did not uncover clear associations. A single study explored the role of rs10462028, a single nucleotide polymorphism (SNP) in the CLOCK gene that is associated with bipolar disorder [24]. While there was no direct link between this SNP and migraine, the study identified a potential interaction between rs10462028, chronic stress (financial difficulties) and migraine [25].

More indirect evidence for a role of circadian regulation in migraine comes from the early activation of the hypothalamus, containing the SCN, including during the earliest premonitory phases of the attack [[26], [27], [28]], and the association between narcolepsy which is a disorder linked to circadian disruption and an increase prevalence of migraine [[29], [30], [31]]. In keeping, people with migraine are considered to be selectively vulnerable to several reported migraine triggers, including stress, the menstrual cycle, bright light, skipping meals and sleep disruption [19], although recent data has proposed that these proposed triggers may be some of the earliest features of an ensuing attack during the premonitory phase [32,33]. Irrespective of their role as triggers or premonitory symptoms, many of these features can influence circadian rhythms and are involved in homeostatic regulation. For example, light is the most powerful zeitgebers for the SCN in mammals which in turn entrains the peripheral molecular clocks. Alternatively, altered feeding schedules is a powerful zeitgeber for the liver, altering the liver's molecular clock independent of the SCN. Experimentally, this property is often used to desynchronize peripheral clocks from the SCN [34], further suggesting that altered feeding patterns and circadian desynchrony may be relevant for migraine. Interestingly, one study has suggested that altered feeding time, eating late at night, could reduce the likelihood of headache [35]. In comparison, circadian disruption is commonly associated with increased attack susceptibility or increasing attack frequency. Jet lag, leading to altered light-dark cycles is a commonly reported migraine trigger [20,36] and night shift work, has been demonstrated to lead to migraine chronification with subsequent reversal to episodic migraine following the return to a normal day shift working patterns [20,21].

## Trigeminovascular system

While migraine is a brain disorder, sensory innervation from the head is relayed via the trigeminal sensory afferents whose cell bodies reside in the trigeminal ganglion [37,38]. These sensory afferents innervate the pain-sensitive meninges and vasculature in the periphery and relay with second order trigeminothalamic projection neurons in the trigeminocervical complex, playing a critical role in migraine-related pain processing (Fig. 1B). In agreement with widespread peripheral molecular clocks, the trigeminal ganglion demonstrates an intrinsic peripheral clock, with rhythmic expression of several hundred genes, including core clock genes [39]. With pharmacogenetic approaches identifying multiple rhythmic genes that encode proteins targeted by drugs used clinically to treat a variety of headache disorders, including migraine (e.g. candesartan, acetaminophen, dihydroergotamine and topiramate) [39]. Upon exposure to chronic nitroglycerin, this circadian gene expression pattern was significantly altered, suggesting that nitroglycerin may act at least in part to disrupt circadian-dependent mechanisms at the level of the peripheral sensory afferents [39]. With mice demonstrating diurnal variations, whereby nitroglycerin evokes a robust mechanical hypersensitivity (allodynia); however, this hypersensitivity was most pronounced during the light phase as compared to the dark (active) phase of the mice [39].

## Migraine therapies with putative actions on circadian genes

While chronotherapy has not yet been widely adopted in migraine, several anti-migraine therapies have direct actions on clock genes/rhythmically expressed proteins. One of the earliest migraine therapies, ergotamine, acting via serotonin receptors including 5-HT_1_, 5-HT_2A_ and 5-HT_2C_, has potent effects on several core clock genes [39]. Enhancing the amplitude of CLOCK, BMAL1, PER3, CRY2 and Rev-erbα/β in a dose-dependent manner. In a proof of principle study exploring the chronotherapeutic potential of ergotamine, Han and colleagues identified a strong effect of ergotamine on the circadian amplitude of fibroblasts and the trigeminal ganglion [39]. Functionally, this circadian action was combined with a time-dependent efficacy on chronic nitroglycerin-induced hypersensitivity (allodynia), with ergotamine significantly reducing hypersensitivity when administered during the day but not at night (active phase) in mice [39]. Ergotamine has largely been superseded by the triptans, specific (5-HT_1B/1D_) receptor agonists, and while there are little data demonstrating a direct effect of triptans on circadian genes, triptans can prevent circadian phase advances to light [40], suggesting a potential impact of triptans on light-induced circadian entrainment, most likely via an action on retinal 5-HT_1B_ receptors [41] modulating light entrainment of the SCN, via the retinohypothalamic tract [23].

More broadly, several migraine and headache-related therapies alter circadian genes. Verapamil, a first line therapeutic for cluster headache, but also used in a small proportion of people with migraine, alters the core clock genes CLOCK, BMAL1, PER1, PER3 and CRY2 [42]. Lithium, again more commonly used in cluster headache and as a mood stabiliser inhibits the GSK3β enzyme, preventing the degradation of Rev-ERBα, a negative regulator of the molecular clock [43]. Finally, propranolol, a preventive treatment for migraine has demonstrated effects on the expression levels of clock genes including BMAL1 [44]. Considering non-specific therapies, the most commonly used anti-migraine therapeutics remain the non-steroidal anti-inflammatory drugs (NSAIDs). The impact of NSAIDs on clock gene expression is well documented and appears to be dependent on the administration time. For example, administration of a commonly used NSAID in rodents (carprofen), resulted in enhanced postoperative surgical pain control, upregulation of the circadian clock genes *Per2* and increased anti-inflammatory cytokine levels when administered during the active phase (night). In comparison, NSAID administration during the rest phase (day) resulted in healing impairment [45], with no benefits on pain control.

## Calcitonin Gene-Related Peptide (CGRP)

Calcitonin gene-related peptide (CGRP) is a key neuropeptide involved in the pathophysiology of migraines following the identification of increased CGRP levels during migraine attacks. Experimentally, CGRP can induce delayed migraine-like attacks in people with migraine [46] and mechanical hypersensitivity and increased migraine-relevant phenotypes in preclinical models of migraine [47,48]. This has led to the development of several CGRP-targeted small molecule antagonists or monoclonal antibodies [[49], [50], [51]] that are approved for the acute (gepants, small molecule CGRP receptor antagonists) or preventive (gepants and monoclonal antibodies targeting CGRP or the CGRP receptor) treatment of migraine [52]. Of particular interest, people with migraine are more likely to demonstrate an extreme chronotype [2] which is associated with when they are most likely to have an attack (e.g. morning larks more commonly have early morning attacks). Following 3-months exposure to erenumab, a monoclonal antibody that targets the CGRP receptor, patients demonstrated a significant reduction in attack frequency and an alteration in their chronotype [53]. Whereby, individual chronotypes normalized from an early morning phenotype to an intermediate phenotype, suggesting that a successful response to CGRP-targeted therapeutics can have a positive impact on an individual's endogenous circadian rhythms and a link between CGRP, circadian rhythms and migraine [53].

## Potential for chronotherapy to optimise therapeutic response to CGRP-targeted therapeutics

This association between therapeutic responsiveness to CGRP-targeted monoclonal antibodies and chronotype normalization [53], the diurnal effect of ergotamine in preclinical models of migraine [39] and the direct impact of several headache-related therapies on clock genes [1] raises a very interesting hypothesis. That is, could chronotherapy enhance the responsiveness to existing therapies including CGRP-targeted therapies. It is established that CGRP levels are increased in people with migraine during an attack [54] and that if CGRP is infused into people with migraine, greater than 60 % of them will develop a delayed CGRP-triggered migraine like attack that is indistinguishable from their normal migraine attacks [46]. As such, it is clear that exogenous CGRP can trigger a delayed migraine-like attack within 1–11 h post-CGRP [46]. As noted previously, the majority of people with migraine demonstrate an increased prevalence of migraine attacks in the early morning [46], often this is the reported time of attack onset; however, given the delayed triggering in human experimental models of migraine [46], it is possible that underlying alterations leading to the onset of the headache, e.g. premonitory symptoms or elevated neuropeptide levels (CGRP in this case) occur hours before the onset of the headache. Considering hormonal rhythms, CGRP levels change significantly across the menstrual cycle [55], suggesting that altering CGRP levels may impact attack susceptibility. While there is very little data reporting circadian alterations in CGRP levels, a diurnal pattern has been reported in males and females following overnight fasting [56], while an alternate study identified a mean circadian acrophase (peak) in the late night (23.14) in healthy males [57] that agrees with nocturnal peaks of 23.25 in normal subjects and 23.14 in hypertensive male and female subjects [58], albeit at lower levels in hypertensive patients.

**Fig. 2:**
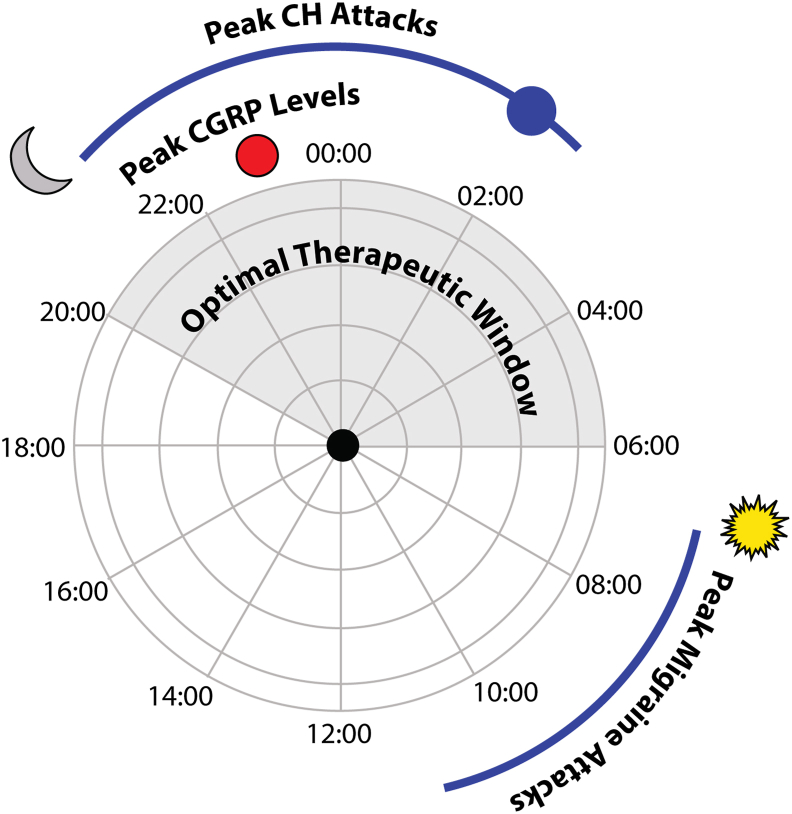
**Potential chronotherapeutic approach for migraine.** Several headache disorders including cluster headache (CH) and migraine demonstrate rhythmic patterns of attack onset. For CH, attacks most commonly occur at approximately 02:00 and for migraine, peak attack onset is reported in the early morning. Calcitonin gene-related peptide (CGRP) has emerged as a key therapeutic target for migraine as its levels increase during migraines and administration of CGRP can trigger delayed migraine-like attacks in people with migraine. Peak CGRP levels in healthy individuals occurs in the late evening (approximately 23:00) suggesting that attempts to block CGRP signalling would be more effective if CGRP-targeted therapies (e.g. gepants) were administered in the late evening.

With this in mind, it raises an interesting hypothesis (Fig. 2) that late night increased CGRP expression may influence the delayed increase in migraine attack occurrence in the early morning, when CGRP levels are lowest [[56], [57], [58]]. It also postulates that a chronotherapeutic approach, whereby administration of CGRP-targeted therapies, including the novel CGRP receptor antagonists the gepants in the evening when CGRP levels are highest [[56], [57], [58]] may prove more effective and increase their clinical efficacy. Such an approach has already been reported for antihypertensive therapies, with bedtime dosing demonstrating better blood pressure control when compared to morning dosing [59]. In agreement, as previously noted, erenumab with an elimination half-life of 28 days, has been shown to be an effective preventive option with contaminant changes in chronotype [53] and sleep quality [60].

## Pituitary adenylate cyclase-activating polypeptide (PACAP)

Pituitary adenylate cyclase-activating polypeptide (PACAP) and PACAP signalling mechanisms are emerging as a potential novel therapeutic target for migraine and cluster headache [61]. Similar to CGRP, PACAP can induce delayed migraine-like attacks in people with migraine [62] and mechanical hypersensitivity and increased nociceptive activity in preclinical models of migraine [63]. Importantly, a recent phase 2 study demonstrated that a monoclonal antibody directed against PACAP significantly reduced migraine frequency [64]. PACAP is a key molecule involved in the regulation of circadian rhythms at several levels. This includes an active role in PER1-2-dependent light-induced phase resetting of the SCN, via PACAP/glutamate co-release from the retinohypothalamic tract [65], which agrees with impaired light entrainment in PAC1 receptor deficient mice [66]. In agreement, loss of vPAC2 receptors abolishes rhythmic activity patterns in approximately half of all SCN neurons [67], resulting in decreased synchrony. Importantly, PACAP is considered to act as a neuromodulator within the SCN, and vPAC2 agonists can act to resynchronize VIP-expressing SCN neurons [67]. While there is only limited evidence for altered circadian expression of PACAP receptors, PAC1 mRNA is differentially expressed in the SCN, with the highest levels during the middle of the light and dark phases of the rodent cycle [68]. More generally, PACAP levels are considered to show a varied circadian expression pattern which is dependent on the brain area and species, for example, PACAP levels are considered to be higher during the night [69]; however, PACAPs role in circadian regulation is intrinsically linked with the external light environment, making it complex to determine specific intrinsic rhythmic patterns. Given the critical role of PACAP in circadian light entrainment [66], it remains to be demonstrated if long term blockade of PACAP signalling will have a positive or negative impact on circadian rhythms and what the subsequent impact on migraine will be.

## Pharmacokinetic and Pharmacodynamics

The molecular clock contributes to a drugs pharmacokinetics and pharmacodynamics, impacting drug absorption, distribution, metabolism and excretion. Alterations of which can play a significant role in regulating the drug bioavailability. In the liver, over 300 genes show circadian rhythmicity in their expression patterns [70], with many of these playing a direct role in drug metabolism. Thus, the precise phase of the circadian cycle is critical for drug administration as it has a robust impact on drug efficacy and toxicity. One important example for migraine is the metabolism of the over the counter medication acetaminophen. Acetaminophen can be metabolised into a potentially toxic by-product called N-acetyl-*p*-benzoquinone imine (NAPQI) which is rapidly detoxified at low doses; however, it has been shown that the levels of NAPQI produced varied by over 50 % depending on the time of drug application and that disrupted circadian rhythms can aggravate acetaminophen-induced liver toxicity in mice [71]. Acetaminophen is metabolised by several enzymes including CYP3A4 which is rhythmically expressed [72] and regulated by BMAL1 [73]. Importantly, approximately 50 % of all drugs [74], including several of the novel CGRP-targeted therapies are metabolised by CYP3A4, suggesting that their metabolism and circulating levels in the body are also likely to follow a rhythmic pattern. Specifically, administration of itraconazole, an inhibitor of CYP3A4 at the same time as atogepant, a small molecule CGRP receptor antagonist, increases the levels of atogepant within the body [75], while co-administration of rifampin, which increases CYP3A4 activity, decreases the levels of atogepant [75]. In agreement, levels of ubrogepant, an alternate small molecule CGRP receptor antagonist are also significantly affected by inhibitors and inducers of CYP3A4 [76], while rimegepant is also metabolised by CYP3A4. Beyond the novel gepant class of therapeutics, CYP3A4 also metabolises several triptans (5-HT_1B/1D_ receptor agonists) [77], highlighting the critical role of drug dose timing to maximise drug availability in the body, promoting efficacy.

## Perspectives

Considering the above discussed role of circadian rhythms in migraine, it stands to reason that circadian hygiene, referring to the maintenance of regular daily habits that align with our circadian rhythms to promote better sleep and health, is likely to play an important role in reducing migraine attack occurrence. It is a commonly used analogy, that “the migraine brain likes you to be boring”. While this is an oversimplified colloquialism, it speaks to the beneficial effects of regular behavioural patterns to maintain homeostasis and reduce attack susceptibility. However, it is clear from several studies that altered rhythmic behaviours can predispose people with migraine to increase attack susceptibility, most notably that a change in typical sleep duration and sleep interruptions, as oppose to too much or too little sleep increases the attack risk [17]. Similarly, many patients report skipping meals or changing working/light-dark patterns (shift work/jet-lag) as attack triggers [[19], [20], [21]]. Thus, good circadian hygiene should be a cornerstone of the migraine conversation. That is, people with migraine should strive to maintain a regular schedule, optimise their sleep environment and minimise exposure to aberrant lighting, especially dim light at night and over exposure to screens which can disrupt natural circadian patterns [78].

The emerging importance of endogenous circadian rhythms in maintaining neurological health is becoming clearer. Chronotherapy, aligns therapeutic interventions with an individual's circadian rhythms to optimise the efficacy and minimise the side effect profile. It is rooted in the understanding that our biological rhythms play a critical role in regulating disease-relevant biological processes and therapeutic targets and offers an alternate approach for personalized interventions, whereby the knowledge of an individual's circadian rhythm (chronotype) informs for example, dose timing to align with peak individual attack occurrence. While this approach is in its infancy in terms of migraine, it offers a scalable and affordable opportunity to optimise therapeutics, considering that the majority of existing drugs are considered to only work in 50 % of the people, 50 % of the time. It is important to note that only limited direct studies exist and future studies should carefully consider age, sex and genetic background as confounding factors which can influence drug metabolism. While the focus of this review is on circadian rhythms, ultradian (<24 h) and infradian (>24 h) rhythms, including hormonal [16] and circannual rhythms [1] can also influence migraine and may impact drug responses. While the focus on chronotype and chronotherapy offers an exciting opportunity, it must be noted that sleep should always be considered as a cofounding factor. Sleep is influenced by both circadian rhythms and sleep homeostasis, meaning that it is complex to isolate chronotype-specific effects. In agreement, it is established that sleep and migraine have a complex relationship [79] and migraine patients commonly present with more disrupted sleep [80] that can be improved with anti-migraine therapeutics [60].

## Author Contribution

PRH wrote the first draft of the manuscript and edited the final version. RF provided feedback and edited the final version.

## Declaration of competing interest

The authors declare the following financial interests/personal relationships which may be considered as potential competing interests:

Philip R Holland reports a relationship with Pfizer Inc that includes: consulting or advisory and speaking and lecture fees. Philip R Holland reports a relationship with Eli Lilly and Company that includes: funding grants. Philip R Holland reports a relationship with Teva Pharmaceuticals Industries Inc that includes: speaking and lecture fees. Philip R Holland reports a relationship with Lundbeck LLC that includes: speaking and lecture fees. Philip R Holland reports a relationship with Novartis Pharmaceuticals that includes: speaking and lecture fees. Philip R Holland reports a relationship with Kallyope that includes: funding grants. Philip R Holland reports a relationship with European Headache Federation that includes: board membership. Philip R Holland reports a relationship with Migraine Science Collaborative that includes: board membership. Philip R Holland reports a relationship with British Association for the Study of Headache that includes: board membership. Rolf Fronczek reports a relationship with TEVA that included: speaking and lecture fees. Rolf Fronczek reports a relationship with Eli Lilly and Company that included: speaking and lecture fees. Rolf Fronczek reports a relationship with Novartis that included: speaking and lecture fees. Rolf Fronczek reports a relationship with Lundbeck LLC that included: speaking and lecture fees. Rolf Fronczek reports a relationship with MedTronic that included: speaking and lecture fees. Rolf Fronczek reports a relationship with Salvia that included: speaking and lecture fees.If there are other authors, they declare that they have no known competing financial interests or personal relationships that could have appeared to influence the work reported in this paper.
